# 16-Isopropyl-5,9-dimethyltetra­cyclo­[10.2.2.0^1,10^.0^4,9^]hexa­dec-15-ene-5,14-dicarboxylic acid ethanol hemisolvate

**DOI:** 10.1107/S1600536809021059

**Published:** 2009-06-06

**Authors:** Hong-Xiao Wang, Shi-Bin Shang, Yan-Bai Yin, Xiao-Ping Rao, Xu Xu

**Affiliations:** a Institute of Chemical Industry of Forest Products, Chinese Academy of Forestry, Nanjing 210042, People’s Republic of China

## Abstract

In the title compound, C_23_H_34_O_4_·0.5C_2_H_6_O, which was isolated from acrylic modified rosin, the endocyclic compound adopts a tetra­cyclo­[10.2.2.01,10.04,9]hexa­decane structure. In the crystal, the components are linked by O—H⋯O and C—H⋯ hydrogen bonds.

## Related literature

The title compound has previously been isolated by solvent extracting (Aldrich, 1971 ▶) and solvent washing (Bicu & Munstata, 2007 ▶) from acrylic modified rosin. 
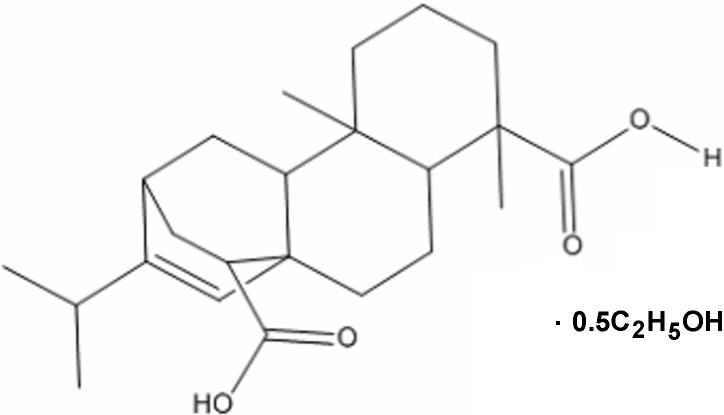


## Experimental

### 

#### Crystal data

C_23_H_34_O_4_·0.5C_2_H_6_O
*M* *_r_* = 397.54
Monoclinic, 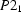

*a* = 12.682 (3) Å
*b* = 12.476 (3) Å
*c* = 14.629 (3) Å
β = 90.12 (3)°
*V* = 2314.6 (9) Å^3^
*Z* = 4
Mo *K*α radiation
μ = 0.08 mm^−1^
*T* = 293 K
0.30 × 0.20 × 0.20 mm

#### Data collection

Enraf–Nonius CAD-4 diffractometer
Absorption correction: ψ scan (*XCAD4*; Harms & Wocadlo, 1995 ▶) *T*_min_ = 0.977, *T*_max_ = 0.985
4620 measured reflections
4413 independent reflections
2780 reflections with *I* > 2σ(*I*)
*R*_int_ = 0.033
3 standard reflections every 200 reflections intensity decay: 1%

#### Refinement

*R*[*F* ^2^ > 2σ(*F* ^2^)] = 0.077
*wR*(*F* ^2^) = 0.194
*S* = 1.02
4413 reflections
480 parameters
24 restraints
H-atom parameters constrained
Δρ_max_ = 0.41 e Å^−3^
Δρ_min_ = −0.47 e Å^−3^

### 

Data collection: *CAD-4 EXPRESS* (Enraf–Nonius, 1989 ▶); cell refinement: *CAD-4 EXPRESS*; data reduction: *XCAD4* (Harms & Wocadlo, 1995 ▶); program(s) used to solve structure: *SHELXS97* (Sheldrick, 2008 ▶); program(s) used to refine structure: *SHELXL97* (Sheldrick, 2008 ▶); molecular graphics: *SHELXTL* (Sheldrick, 2008 ▶); software used to prepare material for publication: *SHELXTL*.

## Supplementary Material

Crystal structure: contains datablocks I, global. DOI: 10.1107/S1600536809021059/at2806sup1.cif

Structure factors: contains datablocks I. DOI: 10.1107/S1600536809021059/at2806Isup2.hkl

Additional supplementary materials:  crystallographic information; 3D view; checkCIF report

## Figures and Tables

**Table 1:** Hydrogen-bond geometry (Å, °)

*D*—H⋯*A*	*D*—H	H⋯*A*	*D*⋯*A*	*D*—H⋯*A*
O1—H1*D*⋯O7^i^	0.82	1.80	2.610 (7)	169
O4—H4*A*⋯O9	0.82	1.77	2.589 (9)	169
O6—H6*A*⋯O3^ii^	0.82	1.86	2.611 (9)	152
O8—H8*C*⋯O2^iii^	0.82	1.90	2.712 (8)	171
O9—H9*B*⋯O5^iv^	0.85	2.23	2.651 (9)	111
C2—H2*C*⋯O6^v^	0.96	2.58	3.443 (12)	150

Symmetry codes: (i) 
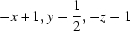
; (ii) 
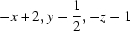
; (iii) 
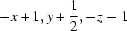
; (iv) 
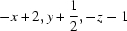
; (v) 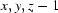.

**Fig. 1.**
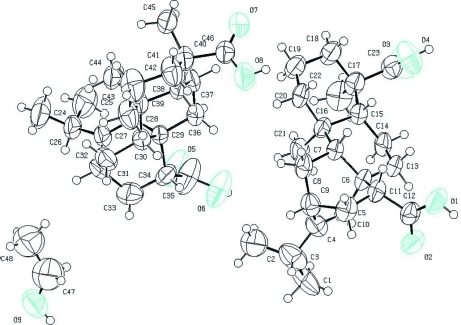
A view of the molecular structure of (I), showing displacement ellipsoids at the 50% probability level.

**Fig. 2.**
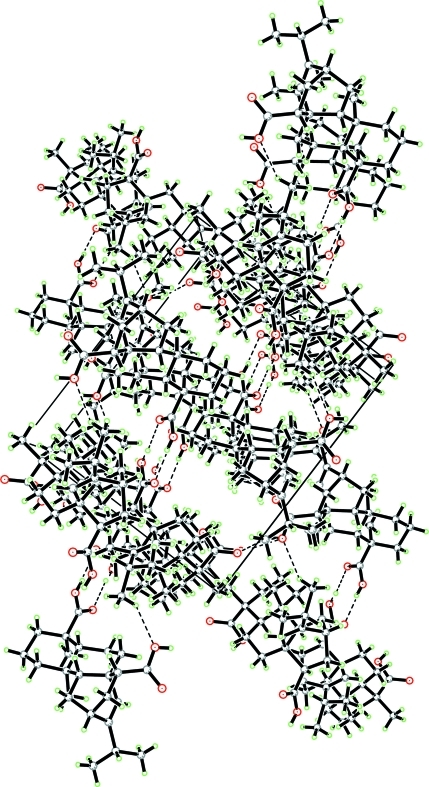
The crystal packing of the title compound. The dash lines indicate hydrogen bonding interactions.

## supplementary crystallographic information

### Comment

Rosin is an abundant and renewable material composed of a series of diterpenic resin acids. It is widely used in production and daily lives in a modified form. Acrylic modified rosin is one of modified products of rosin. Although the title compound has been isolated by solvent extracting (Aldrich, 1971) and solvent washing (Bicu & Munstata, 2007) from acrylic modified rosin, its crystal has not been reported. In this work, we describe the crystal structure of the title compound. The molecular structure is shown in Fig. 1 and the crystal packing in Fig. 2, where the dash lines indicate hydrogen bondsing interactions (Table 1).

### Experimental

The modified rosin (10 g) was dissolved in ethyl alcohol, then 5% sodium hydroxide solution (30 mL) and 2% aqueous sodium chloride solution (500 ml) was added dropwise successively with constant stirring. After dropping the mixture was stirred for another 15 minutes and then filtered. The filtrate was adjusted pH to 3 using 5% hydrochloric acid solution. The title compound was precipitated from the solution. Crystals of the title compound suitable for X-ray diffraction were obtained by slow evaporation of an ethanol solution.

### Refinement

All H atoms were placed geometrically with C—H = 0.93–0.98 Å, O—H = 0.82 Å and included in the refinement in riding motion approximation with *U*_iso_(H) = 1.2 or 1.5*U*_eq_ of the carrier atom. In the absence of significant anomalous dispersion effects Friedel pairs were averaged using MERG 3 in *SHELXL-97*.

### Crystal data

**Table:**

C_23_H_34_O_4_·0.5C_2_H_6_O	*F*(000) = 868
*M**_r_* = 397.54	*D*_x_ = 1.141 Mg m^−^^3^
Monoclinic, *P*2_1_	Melting point: 474 K
Hall symbol: P 2yb	Mo *K*α radiation, λ = 0.71073 Å
*a* = 12.682 (3) Å	Cell parameters from 25 reflections
*b* = 12.476 (3) Å	θ = 9–12°
*c* = 14.629 (3) Å	µ = 0.08 mm^−^^1^
β = 90.12 (3)°	*T* = 293 K
*V* = 2314.6 (9) Å^3^	Block, colourless
*Z* = 4	0.30 × 0.20 × 0.20 mm

### Data collection

**Table:**

Enraf–Nonius CAD-4 diffractometer	2780 reflections with *I* > 2σ(*I*)
Radiation source: fine-focus sealed tube	*R*_int_ = 0.033
graphite	θ_max_ = 25.3°, θ_min_ = 1.4°
ω/2θ scans	*h* = 0→15
Absorption correction: ψ scan (*XCAD4*; Harms & Wocadlo, 1995)	*k* = 0→14
*T*_min_ = 0.977, *T*_max_ = 0.985	*l* = −17→17
4620 measured reflections	3 standard reflections every 200 reflections
4413 independent reflections	intensity decay: 1%

### Refinement

**Table:**

Refinement on *F*^2^	Primary atom site location: structure-invariant direct methods
Least-squares matrix: full	Secondary atom site location: difference Fourier map
*R*[*F*^2^ > 2σ(*F*^2^)] = 0.077	Hydrogen site location: inferred from neighbouring sites
*wR*(*F*^2^) = 0.194	H-atom parameters constrained
*S* = 1.02	*w* = 1/[σ^2^(*F*_o_^2^) + (0.07*P*)^2^ + 2*P*] where *P* = (*F*_o_^2^ + 2*F*_c_^2^)/3
4413 reflections	(Δ/σ)_max_ < 0.001
480 parameters	Δρ_max_ = 0.41 e Å^−^^3^
24 restraints	Δρ_min_ = −0.46 e Å^−^^3^

### Special details

**Table:**

Geometry. All e.s.d.'s (except the e.s.d. in the dihedral angle between two l.s. planes) are estimated using the full covariance matrix. The cell e.s.d.'s are taken into account individually in the estimation of e.s.d.'s in distances, angles and torsion angles; correlations between e.s.d.'s in cell parameters are only used when they are defined by crystal symmetry. An approximate (isotropic) treatment of cell e.s.d.'s is used for estimating e.s.d.'s involving l.s. planes.
Refinement. Refinement of *F*^2^ against ALL reflections. The weighted *R*-factor *wR* and goodness of fit *S* are based on *F*^2^, conventional *R*-factors *R* are based on *F*, with *F* set to zero for negative *F*^2^. The threshold expression of *F*^2^ > σ(*F*^2^) is used only for calculating *R*-factors(gt) *etc*. and is not relevant to the choice of reflections for refinement. *R*-factors based on *F*^2^ are statistically about twice as large as those based on *F*, and *R*- factors based on ALL data will be even larger.

### Fractional atomic coordinates and isotropic or equivalent isotropic displacement parameters (Å^2^)

**Table:**

	*x*	*y*	*z*	*U*_iso_*/*U*_eq_	
O1	0.4273 (5)	0.0876 (5)	−0.8077 (3)	0.102 (2)	
H1D	0.4194	0.0560	−0.7591	0.153*	
O2	0.5275 (4)	−0.0529 (5)	−0.8341 (3)	0.0851 (16)	
O3	0.9581 (5)	0.4395 (7)	−0.8591 (4)	0.126 (2)	
O4	0.7895 (5)	0.4697 (6)	−0.8765 (4)	0.120 (2)	
H4A	0.7994	0.5018	−0.8284	0.180*	
C1	0.7264 (7)	−0.2280 (8)	−1.0584 (6)	0.107	
H1A	0.7431	−0.2911	−1.0929	0.161*	
H1B	0.6911	−0.2481	−1.0030	0.161*	
H1C	0.7903	−0.1903	−1.0436	0.161*	
C2	0.7017 (7)	−0.1389 (9)	−1.2089 (6)	0.106	
H2A	0.6525	−0.0958	−1.2426	0.159*	
H2B	0.7115	−0.2061	−1.2397	0.159*	
H2C	0.7681	−0.1021	−1.2048	0.159*	
C3	0.6586 (7)	−0.1594 (8)	−1.1118 (7)	0.103 (3)	
H3A	0.5918	−0.1980	−1.1194	0.123*	
C4	0.6320 (5)	−0.0537 (6)	−1.0688 (5)	0.072 (2)	
C5	0.6772 (5)	−0.0079 (5)	−0.9964 (4)	0.0544 (15)	
H5A	0.7320	−0.0405	−0.9645	0.065*	
C6	0.6333 (4)	0.1013 (5)	−0.9689 (4)	0.0520 (15)	
C7	0.6353 (4)	0.1774 (5)	−1.0529 (4)	0.0518 (15)	
H7A	0.5896	0.2378	−1.0366	0.062*	
C8	0.5802 (5)	0.1215 (7)	−1.1353 (4)	0.070 (2)	
H8A	0.5220	0.1653	−1.1566	0.084*	
H8B	0.6300	0.1137	−1.1852	0.084*	
C9	0.5399 (5)	0.0127 (6)	−1.1076 (4)	0.0651 (18)	
H9A	0.5074	−0.0240	−1.1598	0.078*	
C10	0.4610 (5)	0.0217 (7)	−1.0277 (4)	0.073 (2)	
H10A	0.3977	0.0586	−1.0479	0.088*	
H10B	0.4411	−0.0493	−1.0069	0.088*	
C11	0.5125 (5)	0.0839 (6)	−0.9494 (4)	0.0606 (17)	
H11A	0.4799	0.1551	−0.9487	0.073*	
C12	0.4906 (5)	0.0343 (6)	−0.8588 (5)	0.0656 (19)	
C13	0.6874 (5)	0.1508 (6)	−0.8859 (4)	0.0612 (17)	
H13A	0.6414	0.2051	−0.8602	0.073*	
H13B	0.6973	0.0956	−0.8399	0.073*	
C14	0.7930 (5)	0.2011 (6)	−0.9069 (4)	0.0654 (18)	
H14A	0.8429	0.1460	−0.9247	0.078*	
H14B	0.8202	0.2368	−0.8528	0.078*	
C15	0.7804 (5)	0.2815 (6)	−0.9837 (4)	0.0555 (15)	
H15A	0.7203	0.3259	−0.9653	0.067*	
C16	0.7447 (5)	0.2279 (5)	−1.0760 (4)	0.0579 (16)	
C17	0.8741 (5)	0.3615 (7)	−0.9916 (4)	0.0689 (19)	
C18	0.8501 (6)	0.4431 (7)	−1.0690 (4)	0.081 (2)	
H18A	0.7924	0.4891	−1.0502	0.098*	
H18B	0.9115	0.4880	−1.0786	0.098*	
C19	0.8213 (6)	0.3891 (7)	−1.1580 (5)	0.078 (2)	
H19A	0.8048	0.4434	−1.2033	0.094*	
H19B	0.8815	0.3487	−1.1799	0.094*	
C20	0.7285 (5)	0.3148 (6)	−1.1481 (4)	0.0656 (18)	
H20A	0.7147	0.2809	−1.2065	0.079*	
H20B	0.6668	0.3567	−1.1320	0.079*	
C21	0.8229 (5)	0.1451 (6)	−1.1122 (5)	0.073 (2)	
H21A	0.7967	0.1155	−1.1684	0.110*	
H21B	0.8313	0.0889	−1.0680	0.110*	
H21C	0.8897	0.1789	−1.1229	0.110*	
C22	0.9823 (5)	0.3088 (9)	−1.0076 (6)	0.094 (3)	
H22A	1.0371	0.3606	−0.9972	0.141*	
H22B	0.9862	0.2831	−1.0693	0.141*	
H22C	0.9911	0.2498	−0.9661	0.141*	
C23	0.8768 (6)	0.4258 (7)	−0.9028 (5)	0.076 (2)	
O5	1.0495 (5)	0.0182 (7)	−0.3837 (4)	0.1285 (17)	
O6	0.9276 (5)	−0.0140 (8)	−0.2842 (4)	0.1285 (17)	
H6A	0.9796	−0.0252	−0.2522	0.193*	
O7	0.6143 (5)	0.5069 (5)	−0.3528 (3)	0.0916 (16)	
O8	0.5194 (5)	0.3646 (5)	−0.3324 (4)	0.0977 (18)	
H8C	0.5094	0.3950	−0.2835	0.147*	
C24	1.0176 (8)	0.1264 (10)	−0.7555 (5)	0.115 (3)	
H24A	1.0768	0.1292	−0.7963	0.172*	
H24B	0.9850	0.1957	−0.7526	0.172*	
H24C	0.9675	0.0748	−0.7776	0.172*	
C25	1.1456 (7)	0.1660 (10)	−0.6261 (6)	0.113 (3)	
H25A	1.1699	0.1391	−0.5683	0.170*	
H25B	1.1207	0.2381	−0.6188	0.170*	
H25C	1.2026	0.1651	−0.6692	0.170*	
C26	1.0545 (6)	0.0943 (7)	−0.6619 (4)	0.079 (2)	
H26A	1.0824	0.0213	−0.6669	0.095*	
C27	0.9606 (5)	0.0895 (6)	−0.5969 (4)	0.0626 (17)	
C28	0.9403 (5)	0.1554 (5)	−0.5295 (4)	0.0570 (16)	
H28A	0.9808	0.2163	−0.5192	0.068*	
C29	0.8485 (5)	0.1274 (5)	−0.4700 (4)	0.0511 (14)	
C30	0.7487 (5)	0.1144 (6)	−0.5314 (4)	0.0608 (16)	
H30A	0.6967	0.0774	−0.4932	0.073*	
C31	0.7718 (6)	0.0385 (6)	−0.6096 (5)	0.078 (2)	
H31A	0.7617	0.0753	−0.6674	0.094*	
H31B	0.7235	−0.0217	−0.6075	0.094*	
C32	0.8852 (6)	−0.0019 (6)	−0.6026 (5)	0.0741 (19)	
H32A	0.9022	−0.0479	−0.6548	0.089*	
C33	0.8950 (7)	−0.0638 (7)	−0.5143 (6)	0.093 (3)	
H33A	0.9661	−0.0915	−0.5080	0.112*	
H33B	0.8466	−0.1240	−0.5148	0.112*	
C34	0.8698 (6)	0.0093 (6)	−0.4343 (4)	0.072 (2)	
H34A	0.8051	−0.0169	−0.4054	0.087*	
C35	0.9563 (8)	0.0079 (12)	−0.3638 (6)	0.1285 (17)	
C36	0.8312 (5)	0.2043 (6)	−0.3913 (4)	0.0625 (17)	
H36A	0.8985	0.2201	−0.3627	0.075*	
H36B	0.7868	0.1702	−0.3459	0.075*	
C37	0.7799 (5)	0.3086 (6)	−0.4215 (4)	0.0610 (17)	
H37A	0.8271	0.3468	−0.4622	0.073*	
H37B	0.7670	0.3536	−0.3686	0.073*	
C38	0.6737 (5)	0.2857 (5)	−0.4717 (4)	0.0555 (15)	
H38A	0.6364	0.2351	−0.4320	0.067*	
C39	0.6950 (5)	0.2234 (6)	−0.5600 (4)	0.0616 (17)	
C40	0.5989 (6)	0.3845 (6)	−0.4794 (4)	0.0693 (19)	
C41	0.4955 (7)	0.3485 (8)	−0.5246 (5)	0.093 (3)	
H41A	0.4581	0.3018	−0.4827	0.112*	
H41B	0.4517	0.4111	−0.5352	0.112*	
C42	0.5107 (7)	0.2896 (9)	−0.6151 (5)	0.108 (3)	
H42A	0.5399	0.3383	−0.6602	0.130*	
H42B	0.4431	0.2644	−0.6376	0.130*	
C43	0.5853 (6)	0.1943 (8)	−0.6017 (5)	0.088 (2)	
H43A	0.5964	0.1601	−0.6604	0.106*	
H43B	0.5513	0.1425	−0.5621	0.106*	
C44	0.7602 (6)	0.2839 (6)	−0.6303 (4)	0.077 (2)	
H44A	0.8272	0.3026	−0.6042	0.115*	
H44B	0.7235	0.3479	−0.6481	0.115*	
H44C	0.7709	0.2393	−0.6830	0.115*	
C45	0.6462 (8)	0.4816 (7)	−0.5321 (5)	0.098 (3)	
H45A	0.5980	0.5408	−0.5297	0.147*	
H45B	0.6579	0.4619	−0.5947	0.147*	
H45C	0.7120	0.5020	−0.5045	0.147*	
C46	0.5767 (6)	0.4226 (7)	−0.3827 (5)	0.0652 (18)	
O9	0.7956 (6)	0.5787 (6)	−0.7267 (4)	0.122 (2)	
H9B	0.7924	0.5357	−0.6818	0.147*	
C48	0.7586 (9)	0.7631 (10)	−0.7751 (8)	0.143	
H48A	0.7287	0.8246	−0.7454	0.215*	
H48B	0.7155	0.7434	−0.8264	0.215*	
H48C	0.8284	0.7801	−0.7959	0.215*	
C47	0.7636 (10)	0.6740 (10)	−0.7108 (8)	0.149	
H47A	0.8064	0.6994	−0.6602	0.179*	
H47B	0.6927	0.6663	−0.6871	0.179*	

### Atomic displacement parameters (Å^2^)

**Table:**

	*U*^11^	*U*^22^	*U*^33^	*U*^12^	*U*^13^	*U*^23^
O1	0.136 (5)	0.096 (4)	0.074 (3)	0.045 (4)	0.051 (3)	0.029 (3)
O2	0.094 (4)	0.084 (4)	0.078 (3)	0.026 (3)	0.024 (3)	0.028 (3)
O3	0.118 (4)	0.157 (6)	0.102 (4)	−0.028 (4)	−0.035 (3)	−0.022 (4)
O4	0.121 (4)	0.140 (5)	0.098 (4)	−0.001 (4)	−0.026 (3)	−0.052 (4)
C1	0.098	0.108	0.114	0.023	−0.071	−0.065
C2	0.106	0.106	0.106	0.000	0.000	0.000
C3	0.076 (5)	0.096 (7)	0.136 (8)	−0.023 (5)	0.016 (5)	−0.043 (6)
C4	0.066 (4)	0.070 (5)	0.081 (5)	−0.013 (4)	0.017 (4)	−0.019 (4)
C5	0.054 (3)	0.054 (4)	0.055 (3)	−0.004 (3)	−0.002 (3)	0.005 (3)
C6	0.049 (3)	0.069 (4)	0.038 (3)	−0.002 (3)	0.003 (2)	0.010 (3)
C7	0.053 (3)	0.064 (4)	0.039 (3)	−0.001 (3)	0.000 (2)	0.002 (3)
C8	0.069 (4)	0.100 (6)	0.041 (3)	−0.015 (4)	−0.009 (3)	−0.006 (4)
C9	0.057 (4)	0.079 (5)	0.059 (4)	−0.012 (4)	0.002 (3)	−0.012 (4)
C10	0.056 (4)	0.097 (6)	0.067 (4)	−0.011 (4)	0.002 (3)	0.003 (4)
C11	0.058 (4)	0.076 (5)	0.048 (3)	0.004 (4)	0.009 (3)	0.008 (3)
C12	0.059 (4)	0.079 (5)	0.059 (4)	−0.008 (4)	0.014 (3)	0.005 (4)
C13	0.080 (4)	0.069 (4)	0.034 (3)	0.000 (4)	0.000 (3)	0.009 (3)
C14	0.073 (4)	0.084 (5)	0.039 (3)	−0.002 (4)	−0.009 (3)	−0.002 (3)
C15	0.057 (3)	0.065 (4)	0.044 (3)	−0.003 (3)	0.002 (3)	0.000 (3)
C16	0.067 (4)	0.064 (4)	0.043 (3)	−0.011 (3)	0.009 (3)	0.006 (3)
C17	0.068 (4)	0.084 (5)	0.055 (4)	−0.014 (4)	−0.003 (3)	0.006 (4)
C18	0.097 (5)	0.092 (6)	0.055 (4)	−0.038 (5)	−0.009 (4)	0.011 (4)
C19	0.092 (5)	0.085 (5)	0.056 (4)	−0.018 (5)	0.001 (4)	0.015 (4)
C20	0.073 (4)	0.089 (5)	0.035 (3)	0.000 (4)	−0.007 (3)	0.007 (3)
C21	0.070 (4)	0.084 (5)	0.066 (4)	−0.011 (4)	0.029 (3)	−0.010 (4)
C22	0.054 (4)	0.131 (8)	0.097 (5)	−0.010 (5)	−0.007 (4)	−0.002 (6)
C23	0.083 (4)	0.086 (5)	0.058 (4)	−0.015 (4)	−0.019 (3)	0.004 (4)
O5	0.106 (3)	0.208 (5)	0.071 (2)	0.011 (4)	0.000 (2)	0.044 (3)
O6	0.106 (3)	0.208 (5)	0.071 (2)	0.011 (4)	0.000 (2)	0.044 (3)
O7	0.124 (4)	0.083 (4)	0.068 (3)	−0.001 (4)	0.026 (3)	−0.007 (3)
O8	0.112 (4)	0.112 (5)	0.070 (3)	−0.011 (4)	0.026 (3)	−0.017 (3)
C24	0.139 (8)	0.146 (9)	0.058 (4)	0.037 (7)	0.027 (5)	0.015 (6)
C25	0.095 (6)	0.149 (10)	0.096 (6)	0.010 (7)	0.036 (5)	0.009 (7)
C26	0.105 (6)	0.079 (5)	0.054 (4)	0.021 (5)	0.028 (4)	−0.001 (4)
C27	0.076 (4)	0.062 (4)	0.050 (3)	0.012 (4)	0.010 (3)	−0.008 (3)
C28	0.061 (4)	0.060 (4)	0.049 (3)	0.001 (3)	0.006 (3)	−0.003 (3)
C29	0.061 (4)	0.051 (4)	0.041 (3)	0.006 (3)	0.004 (3)	−0.001 (3)
C30	0.068 (4)	0.065 (4)	0.049 (3)	0.002 (3)	0.000 (3)	−0.005 (3)
C31	0.092 (5)	0.069 (5)	0.074 (5)	−0.005 (4)	−0.008 (4)	−0.020 (4)
C32	0.098 (5)	0.052 (4)	0.072 (4)	0.013 (4)	0.006 (4)	−0.013 (4)
C33	0.107 (6)	0.070 (5)	0.103 (6)	0.019 (5)	0.001 (5)	0.009 (5)
C34	0.091 (5)	0.070 (5)	0.057 (4)	0.005 (4)	0.010 (3)	0.023 (4)
C35	0.106 (3)	0.208 (5)	0.071 (2)	0.011 (4)	0.000 (2)	0.044 (3)
C36	0.070 (4)	0.076 (5)	0.042 (3)	−0.001 (4)	0.000 (3)	−0.005 (3)
C37	0.069 (4)	0.073 (5)	0.041 (3)	−0.010 (4)	0.004 (3)	−0.022 (3)
C38	0.065 (4)	0.061 (4)	0.040 (3)	0.004 (3)	0.001 (3)	0.001 (3)
C39	0.072 (4)	0.072 (4)	0.041 (3)	0.001 (4)	−0.002 (3)	−0.005 (3)
C40	0.080 (5)	0.082 (5)	0.046 (3)	0.020 (4)	0.003 (3)	−0.006 (4)
C41	0.097 (5)	0.118 (7)	0.064 (5)	0.033 (5)	−0.016 (4)	−0.018 (5)
C42	0.096 (6)	0.151 (9)	0.078 (5)	0.042 (7)	−0.030 (4)	−0.012 (6)
C43	0.091 (5)	0.112 (7)	0.061 (4)	0.015 (5)	−0.021 (4)	−0.022 (5)
C44	0.106 (5)	0.080 (5)	0.045 (3)	0.029 (5)	0.014 (3)	0.003 (4)
C45	0.158 (8)	0.077 (6)	0.060 (4)	0.038 (6)	0.021 (5)	0.009 (4)
C46	0.069 (4)	0.074 (5)	0.053 (4)	0.006 (4)	0.006 (3)	−0.001 (4)
O9	0.172 (6)	0.099 (5)	0.096 (4)	0.034 (5)	−0.003 (4)	−0.020 (4)
C48	0.143	0.143	0.143	0.000	0.000	0.000
C47	0.149	0.149	0.149	0.000	0.000	0.000

### Geometric parameters (Å, °)

**Table:**

O1—C12	1.284 (8)	O8—C46	1.262 (9)
O1—H1D	0.8200	O8—H8C	0.8200
O2—C12	1.237 (9)	C24—C26	1.501 (10)
O3—C23	1.224 (8)	C24—H24A	0.9600
O4—C23	1.293 (9)	C24—H24B	0.9600
O4—H4A	0.8200	C24—H24C	0.9600
C1—C3	1.442 (12)	C25—C26	1.551 (12)
C1—H1A	0.9600	C25—H25A	0.9600
C1—H1B	0.9600	C25—H25B	0.9600
C1—H1C	0.9600	C25—H25C	0.9600
C2—C3	1.545 (12)	C26—C27	1.527 (9)
C2—H2A	0.9600	C26—H26A	0.9800
C2—H2B	0.9600	C27—C28	1.309 (8)
C2—H2C	0.9600	C27—C32	1.490 (10)
C3—C4	1.499 (11)	C28—C29	1.496 (8)
C3—H3A	0.9800	C28—H28A	0.9300
C4—C5	1.333 (9)	C29—C36	1.516 (8)
C4—C9	1.540 (10)	C29—C30	1.558 (8)
C5—C6	1.526 (9)	C29—C34	1.586 (9)
C5—H5A	0.9300	C30—C31	1.514 (9)
C6—C13	1.525 (8)	C30—C39	1.576 (10)
C6—C7	1.552 (8)	C30—H30A	0.9800
C6—C11	1.574 (8)	C31—C32	1.527 (10)
C7—C8	1.558 (8)	C31—H31A	0.9700
C7—C16	1.562 (8)	C31—H31B	0.9700
C7—H7A	0.9800	C32—C33	1.510 (10)
C8—C9	1.507 (10)	C32—H32A	0.9800
C8—H8A	0.9700	C33—C34	1.518 (11)
C8—H8B	0.9700	C33—H33A	0.9700
C9—C10	1.544 (9)	C33—H33B	0.9700
C9—H9A	0.9800	C34—C35	1.504 (12)
C10—C11	1.528 (9)	C34—H34A	0.9800
C10—H10A	0.9700	C36—C37	1.520 (10)
C10—H10B	0.9700	C36—H36A	0.9700
C11—C12	1.490 (9)	C36—H36B	0.9700
C11—H11A	0.9800	C37—C38	1.559 (8)
C13—C14	1.512 (9)	C37—H37A	0.9700
C13—H13A	0.9700	C37—H37B	0.9700
C13—H13B	0.9700	C38—C39	1.532 (8)
C14—C15	1.514 (9)	C38—C40	1.560 (9)
C14—H14A	0.9700	C38—H38A	0.9800
C14—H14B	0.9700	C39—C44	1.522 (10)
C15—C17	1.556 (9)	C39—C43	1.561 (10)
C15—C16	1.573 (8)	C40—C46	1.520 (9)
C15—H15A	0.9800	C40—C41	1.534 (11)
C16—C20	1.526 (9)	C40—C45	1.557 (11)
C16—C21	1.527 (9)	C41—C42	1.527 (11)
C17—C23	1.527 (10)	C41—H41A	0.9700
C17—C22	1.540 (10)	C41—H41B	0.9700
C17—C18	1.553 (10)	C42—C43	1.532 (12)
C18—C19	1.510 (9)	C42—H42A	0.9700
C18—H18A	0.9700	C42—H42B	0.9700
C18—H18B	0.9700	C43—H43A	0.9700
C19—C20	1.505 (10)	C43—H43B	0.9700
C19—H19A	0.9700	C44—H44A	0.9600
C19—H19B	0.9700	C44—H44B	0.9600
C20—H20A	0.9700	C44—H44C	0.9600
C20—H20B	0.9700	C45—H45A	0.9600
C21—H21A	0.9600	C45—H45B	0.9600
C21—H21B	0.9600	C45—H45C	0.9600
C21—H21C	0.9600	O9—C47	1.278 (11)
C22—H22A	0.9600	O9—H9B	0.8499
C22—H22B	0.9600	C48—C47	1.458 (9)
C22—H22C	0.9600	C48—H48A	0.9600
O5—C35	1.224 (10)	C48—H48B	0.9600
O6—C35	1.252 (10)	C48—H48C	0.9600
O6—H6A	0.8200	C47—H47A	0.9700
O7—C46	1.234 (9)	C47—H47B	0.9700
			
C12—O1—H1D	109.5	H24B—C24—H24C	109.5
C23—O4—H4A	109.5	C26—C25—H25A	109.5
C3—C1—H1A	109.5	C26—C25—H25B	109.5
C3—C1—H1B	109.5	H25A—C25—H25B	109.5
H1A—C1—H1B	109.5	C26—C25—H25C	109.5
C3—C1—H1C	109.5	H25A—C25—H25C	109.5
H1A—C1—H1C	109.5	H25B—C25—H25C	109.5
H1B—C1—H1C	109.5	C24—C26—C27	109.7 (7)
C3—C2—H2A	109.5	C24—C26—C25	112.6 (8)
C3—C2—H2B	109.5	C27—C26—C25	113.2 (6)
H2A—C2—H2B	109.5	C24—C26—H26A	107.0
C3—C2—H2C	109.5	C27—C26—H26A	107.0
H2A—C2—H2C	109.5	C25—C26—H26A	107.0
H2B—C2—H2C	109.5	C28—C27—C32	113.3 (6)
C1—C3—C4	115.4 (8)	C28—C27—C26	126.8 (7)
C1—C3—C2	112.6 (8)	C32—C27—C26	119.8 (6)
C4—C3—C2	108.7 (8)	C27—C28—C29	116.5 (6)
C1—C3—H3A	106.5	C27—C28—H28A	121.8
C4—C3—H3A	106.5	C29—C28—H28A	121.8
C2—C3—H3A	106.5	C28—C29—C36	114.1 (5)
C5—C4—C3	127.9 (8)	C28—C29—C30	108.7 (5)
C5—C4—C9	112.7 (6)	C36—C29—C30	112.6 (5)
C3—C4—C9	119.3 (7)	C28—C29—C34	106.0 (5)
C4—C5—C6	115.8 (6)	C36—C29—C34	111.2 (5)
C4—C5—H5A	122.1	C30—C29—C34	103.3 (5)
C6—C5—H5A	122.1	C31—C30—C29	110.0 (5)
C13—C6—C5	114.1 (5)	C31—C30—C39	115.0 (5)
C13—C6—C7	112.0 (5)	C29—C30—C39	114.4 (5)
C5—C6—C7	109.4 (4)	C31—C30—H30A	105.5
C13—C6—C11	110.3 (5)	C29—C30—H30A	105.5
C5—C6—C11	106.3 (5)	C39—C30—H30A	105.5
C7—C6—C11	104.2 (5)	C30—C31—C32	109.8 (6)
C6—C7—C8	109.3 (5)	C30—C31—H31A	109.7
C6—C7—C16	115.7 (5)	C32—C31—H31A	109.7
C8—C7—C16	114.2 (5)	C30—C31—H31B	109.7
C6—C7—H7A	105.6	C32—C31—H31B	109.7
C8—C7—H7A	105.6	H31A—C31—H31B	108.2
C16—C7—H7A	105.6	C27—C32—C33	107.0 (6)
C9—C8—C7	110.3 (5)	C27—C32—C31	110.8 (6)
C9—C8—H8A	109.6	C33—C32—C31	107.6 (6)
C7—C8—H8A	109.6	C27—C32—H32A	110.5
C9—C8—H8B	109.6	C33—C32—H32A	110.5
C7—C8—H8B	109.6	C31—C32—H32A	110.5
H8A—C8—H8B	108.1	C32—C33—C34	109.6 (6)
C8—C9—C4	109.0 (5)	C32—C33—H33A	109.8
C8—C9—C10	111.1 (6)	C34—C33—H33A	109.8
C4—C9—C10	104.7 (6)	C32—C33—H33B	109.8
C8—C9—H9A	110.6	C34—C33—H33B	109.8
C4—C9—H9A	110.6	H33A—C33—H33B	108.2
C10—C9—H9A	110.6	C35—C34—C33	111.5 (8)
C11—C10—C9	109.1 (5)	C35—C34—C29	111.1 (8)
C11—C10—H10A	109.9	C33—C34—C29	109.9 (5)
C9—C10—H10A	109.9	C35—C34—H34A	108.1
C11—C10—H10B	109.9	C33—C34—H34A	108.1
C9—C10—H10B	109.9	C29—C34—H34A	108.1
H10A—C10—H10B	108.3	O5—C35—O6	121.8 (9)
C12—C11—C10	112.1 (6)	O5—C35—C34	122.6 (8)
C12—C11—C6	113.7 (5)	O6—C35—C34	115.3 (9)
C10—C11—C6	110.4 (5)	C29—C36—C37	112.5 (5)
C12—C11—H11A	106.7	C29—C36—H36A	109.1
C10—C11—H11A	106.7	C37—C36—H36A	109.1
C6—C11—H11A	106.7	C29—C36—H36B	109.1
O2—C12—O1	121.5 (6)	C37—C36—H36B	109.1
O2—C12—C11	123.6 (7)	H36A—C36—H36B	107.8
O1—C12—C11	114.8 (7)	C36—C37—C38	110.4 (5)
C14—C13—C6	113.8 (5)	C36—C37—H37A	109.6
C14—C13—H13A	108.8	C38—C37—H37A	109.6
C6—C13—H13A	108.8	C36—C37—H37B	109.6
C14—C13—H13B	108.8	C38—C37—H37B	109.6
C6—C13—H13B	108.8	H37A—C37—H37B	108.1
H13A—C13—H13B	107.7	C39—C38—C37	109.6 (5)
C13—C14—C15	109.5 (5)	C39—C38—C40	116.6 (5)
C13—C14—H14A	109.8	C37—C38—C40	114.5 (6)
C15—C14—H14A	109.8	C39—C38—H38A	104.9
C13—C14—H14B	109.8	C37—C38—H38A	104.9
C15—C14—H14B	109.8	C40—C38—H38A	104.9
H14A—C14—H14B	108.2	C44—C39—C38	114.5 (6)
C14—C15—C17	113.6 (5)	C44—C39—C43	109.6 (6)
C14—C15—C16	112.7 (5)	C38—C39—C43	106.8 (5)
C17—C15—C16	115.3 (5)	C44—C39—C30	111.9 (6)
C14—C15—H15A	104.6	C38—C39—C30	106.9 (5)
C17—C15—H15A	104.6	C43—C39—C30	106.7 (6)
C16—C15—H15A	104.6	C46—C40—C41	109.5 (6)
C20—C16—C21	109.1 (5)	C46—C40—C45	106.9 (6)
C20—C16—C7	108.6 (5)	C41—C40—C45	110.1 (6)
C21—C16—C7	112.3 (6)	C46—C40—C38	107.0 (5)
C20—C16—C15	109.2 (5)	C41—C40—C38	108.6 (6)
C21—C16—C15	113.5 (5)	C45—C40—C38	114.6 (6)
C7—C16—C15	103.9 (4)	C42—C41—C40	113.9 (7)
C23—C17—C22	109.6 (6)	C42—C41—H41A	108.8
C23—C17—C18	106.3 (6)	C40—C41—H41A	108.8
C22—C17—C18	110.0 (6)	C42—C41—H41B	108.8
C23—C17—C15	106.8 (5)	C40—C41—H41B	108.8
C22—C17—C15	114.7 (7)	H41A—C41—H41B	107.7
C18—C17—C15	109.0 (5)	C41—C42—C43	110.0 (6)
C19—C18—C17	112.5 (7)	C41—C42—H42A	109.7
C19—C18—H18A	109.1	C43—C42—H42A	109.7
C17—C18—H18A	109.1	C41—C42—H42B	109.7
C19—C18—H18B	109.1	C43—C42—H42B	109.7
C17—C18—H18B	109.1	H42A—C42—H42B	108.2
H18A—C18—H18B	107.8	C42—C43—C39	114.8 (7)
C20—C19—C18	112.3 (6)	C42—C43—H43A	108.6
C20—C19—H19A	109.1	C39—C43—H43A	108.6
C18—C19—H19A	109.1	C42—C43—H43B	108.6
C20—C19—H19B	109.1	C39—C43—H43B	108.6
C18—C19—H19B	109.1	H43A—C43—H43B	107.5
H19A—C19—H19B	107.9	C39—C44—H44A	109.5
C19—C20—C16	113.6 (5)	C39—C44—H44B	109.5
C19—C20—H20A	108.8	H44A—C44—H44B	109.5
C16—C20—H20A	108.8	C39—C44—H44C	109.5
C19—C20—H20B	108.8	H44A—C44—H44C	109.5
C16—C20—H20B	108.8	H44B—C44—H44C	109.5
H20A—C20—H20B	107.7	C40—C45—H45A	109.5
C16—C21—H21A	109.5	C40—C45—H45B	109.5
C16—C21—H21B	109.5	H45A—C45—H45B	109.5
H21A—C21—H21B	109.5	C40—C45—H45C	109.5
C16—C21—H21C	109.5	H45A—C45—H45C	109.5
H21A—C21—H21C	109.5	H45B—C45—H45C	109.5
H21B—C21—H21C	109.5	O7—C46—O8	120.4 (7)
C17—C22—H22A	109.5	O7—C46—C40	121.6 (7)
C17—C22—H22B	109.5	O8—C46—C40	118.1 (7)
H22A—C22—H22B	109.5	C47—O9—H9B	115.5
C17—C22—H22C	109.5	C47—C48—H48A	109.5
H22A—C22—H22C	109.5	C47—C48—H48B	109.5
H22B—C22—H22C	109.5	H48A—C48—H48B	109.5
O3—C23—O4	120.4 (8)	C47—C48—H48C	109.5
O3—C23—C17	122.3 (8)	H48A—C48—H48C	109.5
O4—C23—C17	117.2 (6)	H48B—C48—H48C	109.5
C35—O6—H6A	109.5	O9—C47—C48	127.2 (12)
C46—O8—H8C	109.5	O9—C47—H47A	105.5
C26—C24—H24A	109.5	C48—C47—H47A	105.5
C26—C24—H24B	109.5	O9—C47—H47B	105.5
H24A—C24—H24B	109.5	C48—C47—H47B	105.5
C26—C24—H24C	109.5	H47A—C47—H47B	106.1
H24A—C24—H24C	109.5		
			
C1—C3—C4—C5	12.9 (12)	C24—C26—C27—C28	−109.9 (9)
C2—C3—C4—C5	−114.6 (9)	C25—C26—C27—C28	16.8 (11)
C1—C3—C4—C9	−164.8 (7)	C24—C26—C27—C32	74.9 (9)
C2—C3—C4—C9	67.6 (9)	C25—C26—C27—C32	−158.4 (7)
C3—C4—C5—C6	179.5 (7)	C32—C27—C28—C29	1.1 (9)
C9—C4—C5—C6	−2.7 (8)	C26—C27—C28—C29	−174.4 (6)
C4—C5—C6—C13	−179.9 (6)	C27—C28—C29—C36	177.9 (6)
C4—C5—C6—C7	−53.6 (7)	C27—C28—C29—C30	−55.4 (7)
C4—C5—C6—C11	58.3 (6)	C27—C28—C29—C34	55.1 (7)
C13—C6—C7—C8	179.3 (5)	C28—C29—C30—C31	51.4 (7)
C5—C6—C7—C8	51.8 (6)	C36—C29—C30—C31	178.9 (6)
C11—C6—C7—C8	−61.5 (6)	C34—C29—C30—C31	−61.0 (7)
C13—C6—C7—C16	48.7 (7)	C28—C29—C30—C39	−79.9 (6)
C5—C6—C7—C16	−78.8 (6)	C36—C29—C30—C39	47.6 (7)
C11—C6—C7—C16	168.0 (5)	C34—C29—C30—C39	167.8 (5)
C6—C7—C8—C9	1.5 (7)	C29—C30—C31—C32	0.6 (8)
C16—C7—C8—C9	132.9 (6)	C39—C30—C31—C32	131.5 (6)
C7—C8—C9—C4	−56.1 (7)	C28—C27—C32—C33	−61.3 (8)
C7—C8—C9—C10	58.8 (7)	C26—C27—C32—C33	114.6 (7)
C5—C4—C9—C8	59.5 (7)	C28—C27—C32—C31	55.7 (8)
C3—C4—C9—C8	−122.5 (7)	C26—C27—C32—C31	−128.4 (7)
C5—C4—C9—C10	−59.5 (7)	C30—C31—C32—C27	−54.1 (8)
C3—C4—C9—C10	118.6 (7)	C30—C31—C32—C33	62.5 (8)
C8—C9—C10—C11	−54.0 (8)	C27—C32—C33—C34	59.0 (8)
C4—C9—C10—C11	63.5 (7)	C31—C32—C33—C34	−60.1 (8)
C9—C10—C11—C12	−137.2 (6)	C32—C33—C34—C35	−127.1 (8)
C9—C10—C11—C6	−9.3 (8)	C32—C33—C34—C29	−3.4 (9)
C13—C6—C11—C12	−46.0 (8)	C28—C29—C34—C35	72.8 (7)
C5—C6—C11—C12	78.1 (7)	C36—C29—C34—C35	−51.8 (8)
C7—C6—C11—C12	−166.4 (6)	C30—C29—C34—C35	−172.9 (6)
C13—C6—C11—C10	−172.9 (6)	C28—C29—C34—C33	−51.1 (7)
C5—C6—C11—C10	−48.8 (7)	C36—C29—C34—C33	−175.7 (6)
C7—C6—C11—C10	66.7 (7)	C30—C29—C34—C33	63.2 (7)
C10—C11—C12—O2	69.1 (9)	C33—C34—C35—O5	48.2 (16)
C6—C11—C12—O2	−57.0 (9)	C29—C34—C35—O5	−74.8 (15)
C10—C11—C12—O1	−109.0 (7)	C33—C34—C35—O6	−125.7 (11)
C6—C11—C12—O1	124.9 (7)	C29—C34—C35—O6	111.3 (12)
C5—C6—C13—C14	78.2 (7)	C28—C29—C36—C37	76.8 (7)
C7—C6—C13—C14	−46.7 (8)	C30—C29—C36—C37	−47.8 (7)
C11—C6—C13—C14	−162.3 (6)	C34—C29—C36—C37	−163.3 (5)
C6—C13—C14—C15	54.2 (8)	C29—C36—C37—C38	56.3 (7)
C13—C14—C15—C17	162.8 (6)	C36—C37—C38—C39	−64.4 (6)
C13—C14—C15—C16	−63.8 (7)	C36—C37—C38—C40	162.4 (5)
C6—C7—C16—C20	−169.8 (5)	C37—C38—C39—C44	−63.5 (7)
C8—C7—C16—C20	62.0 (7)	C40—C38—C39—C44	68.6 (7)
C6—C7—C16—C21	69.4 (7)	C37—C38—C39—C43	175.0 (6)
C8—C7—C16—C21	−58.8 (7)	C40—C38—C39—C43	−52.9 (8)
C6—C7—C16—C15	−53.7 (7)	C37—C38—C39—C30	61.0 (7)
C8—C7—C16—C15	178.1 (6)	C40—C38—C39—C30	−166.8 (5)
C14—C15—C16—C20	177.2 (5)	C31—C30—C39—C44	−56.5 (8)
C17—C15—C16—C20	−50.2 (7)	C29—C30—C39—C44	72.3 (7)
C14—C15—C16—C21	−60.8 (7)	C31—C30—C39—C38	177.4 (6)
C17—C15—C16—C21	71.8 (7)	C29—C30—C39—C38	−53.9 (7)
C14—C15—C16—C7	61.5 (6)	C31—C30—C39—C43	63.4 (7)
C17—C15—C16—C7	−165.9 (6)	C29—C30—C39—C43	−167.9 (5)
C14—C15—C17—C23	−62.8 (8)	C39—C38—C40—C46	171.7 (6)
C16—C15—C17—C23	165.0 (6)	C37—C38—C40—C46	−58.4 (7)
C14—C15—C17—C22	58.9 (8)	C39—C38—C40—C41	53.6 (8)
C16—C15—C17—C22	−73.3 (7)	C37—C38—C40—C41	−176.5 (5)
C14—C15—C17—C18	−177.3 (6)	C39—C38—C40—C45	−70.0 (8)
C16—C15—C17—C18	50.6 (8)	C37—C38—C40—C45	59.9 (7)
C23—C17—C18—C19	−167.4 (6)	C46—C40—C41—C42	−169.3 (8)
C22—C17—C18—C19	74.0 (8)	C45—C40—C41—C42	73.5 (9)
C15—C17—C18—C19	−52.6 (8)	C38—C40—C41—C42	−52.7 (9)
C17—C18—C19—C20	56.9 (9)	C40—C41—C42—C43	55.0 (11)
C18—C19—C20—C16	−57.0 (9)	C41—C42—C43—C39	−55.8 (10)
C21—C16—C20—C19	−72.7 (7)	C44—C39—C43—C42	−71.1 (8)
C7—C16—C20—C19	164.5 (6)	C38—C39—C43—C42	53.5 (8)
C15—C16—C20—C19	51.9 (7)	C30—C39—C43—C42	167.6 (6)
C22—C17—C23—O3	6.9 (11)	C41—C40—C46—O7	−133.9 (8)
C18—C17—C23—O3	−112.0 (9)	C45—C40—C46—O7	−14.6 (9)
C15—C17—C23—O3	131.7 (8)	C38—C40—C46—O7	108.6 (8)
C22—C17—C23—O4	−175.7 (8)	C41—C40—C46—O8	47.1 (9)
C18—C17—C23—O4	65.5 (9)	C45—C40—C46—O8	166.4 (7)
C15—C17—C23—O4	−50.9 (9)	C38—C40—C46—O8	−70.5 (8)

### Hydrogen-bond geometry (Å, °)

**Table:**

*D*—H···*A*	*D*—H	H···*A*	*D*···*A*	*D*—H···*A*
C15—H15A···O4	0.98	2.38	2.824 (10)	107
C18—H18A···O4	0.97	2.55	2.939 (8)	104
C33—H33A···O5	0.97	2.51	2.919 (11)	105
C41—H41A···O8	0.97	2.46	2.834 (9)	103
O1—H1D···O7^i^	0.82	1.80	2.610 (7)	169
O4—H4A···O9	0.82	1.77	2.589 (9)	169
O6—H6A···O3^ii^	0.82	1.86	2.611 (9)	152
O8—H8C···O2^iii^	0.82	1.90	2.712 (8)	171
O9—H9B···O5^iv^	0.85	2.23	2.651 (9)	111
C2—H2C···O6^v^	0.96	2.58	3.443 (12)	150

Symmetry codes: (i) −*x*+1, *y*−1/2, −*z*−1; (ii) −*x*+2, *y*−1/2, −*z*−1; (iii) −*x*+1, *y*+1/2, −*z*−1; (iv) −*x*+2, *y*+1/2, −*z*−1; (v) *x*, *y*, *z*−1.

## References

[bb1] Aldrich, P. H. (1971). US Patent No. 3 562 243.

[bb2] Bicu, I. & Munstata, F. (2007). *J. Polym. Sci. Part A Polym. Chem.* **45**, 5979–5990.

[bb3] Enraf–Nonius (1989). *CAD-4 Software* Enraf–Nonius, Delft, The Netherlands.

[bb4] Harms, K. & Wocadlo, S. (1995). *XCAD4* University of Marburg, Germany.

[bb5] Sheldrick, G. M. (2008). *Acta Cryst.* A**64**, 112–122. 10.1107/S0108767307043930 18156677

